# A Comprehensive Comparison of Bovine and Porcine Decellularized Pericardia: New Insights for Surgical Applications

**DOI:** 10.3390/biom10030371

**Published:** 2020-02-28

**Authors:** Sabra Zouhair, Eleonora Dal Sasso, Sugat R. Tuladhar, Catia Fidalgo, Luca Vedovelli, Andrea Filippi, Giulia Borile, Andrea Bagno, Massimo Marchesan, Giorgio De Rossi, Dario Gregori, Willem F. Wolkers, Filippo Romanato, Sotirios Korossis, Gino Gerosa, Laura Iop

**Affiliations:** 1 Cardiovascular Regenerative Medicine, Department of Cardiac Thoracic Vascular Sciences and Public Health, University of Padua, I-35128 Padua, Italy; 2 Biostatistics, Department of Cardiac Thoracic Vascular Sciences and Public Health, University of Padua, I-35128 Padua, Italy; 3 Department of Physics and Astronomy "G. Galilei," University of Padua, I-35131 Padua, Italy; 4 Fondazione Bruno Kessler, I-38123 Trento, Italy; 5 Institute of Pediatric Research Città della Speranza, I-35127 Padua, Italy; 6 Department of Biomedical Sciences, University of Padua, I-35131 Padua, Italy; 7 Department of Industrial Engineering, University of Padua, I-35131 Padua, Italy; 8 L.I.F.E.L.A.B. Program, Consorzio per la Ricerca Sanitaria (CORIS), Veneto Region, I-35127 Padua, Italy; 9 ULSS 3 Serenissima, Mestre, I-30174 Venice, Italy; 10 Institute of Multiphase Processes, Leibniz Universität Hannover, D-30167 Hannover, Germany; 11 Laboratory for Nanofabrication of Nanodevices, I-35127 Padua, Italy; 12 Department of Cardiothoracic, Transplantation and Vascular Surgery, Hannover Medical School, D-30625 Hannover, Germany; 13 Lower Saxony Centre for Biomedical Engineering Implant Research and Development, Hannover Medical School, D-30625 Hannover, Germany; 14 Centre for Biological Engineering, Wolfson School of Mechanical, Electrical and Manufacturing Engineering, Loughborough University, Loughborough LE11 3TU, Leicestershire, UK

**Keywords:** bovine pericardium, porcine pericardium, decellularization, surgery, surgical replacements, tissue engineering

## Abstract

Xenogeneic pericardium-based substitutes are employed for several surgical indications after chemical shielding, limiting their biocompatibility and therapeutic durability. Adverse responses to these replacements might be prevented by tissue decellularization, ideally removing cells and preserving the original extracellular matrix (ECM). The aim of this study was to compare the mostly applied pericardia in clinics, i.e., bovine and porcine tissues, after their decellularization, and obtain new insights for their possible surgical use. Bovine and porcine pericardia were submitted to TRICOL decellularization, based on osmotic shock, detergents and nuclease treatment. TRICOL procedure resulted in being effective in cell removal and preservation of ECM architecture of both species’ scaffolds. Collagen and elastin were retained but glycosaminoglycans were reduced, significantly for bovine scaffolds. Tissue hydration was varied by decellularization, with a rise for bovine pericardia and a decrease for porcine ones. TRICOL significantly increased porcine pericardial thickness, while a non-significant reduction was observed for the bovine counterpart. The protein secondary structure and thermal denaturation profile of both species’ scaffolds were unaltered. Both pericardial tissues showed augmented biomechanical compliance after decellularization. The ECM bioactivity of bovine and porcine pericardia was unaffected by decellularization, sustaining viability and proliferation of human mesenchymal stem cells and endothelial cells. In conclusion, decellularized bovine and porcine pericardia demonstrate possessing the characteristics that are suitable for the creation of novel scaffolds for reconstruction or replacement: differences in water content, thickness and glycosaminoglycans might influence some of their biomechanical properties and, hence, their indication for surgical use.

## 1. Introduction

Among biological tissues, pericardium has shown large versatility as a replacement alternative in several surgical fields [1,2,3,4,5,6,7,8]. Besides bioprosthetic valve formulation [9], glutaraldehyde (GA)-treated bovine, porcine, equine, and human pericardia are used for repair or substitution in the surgery of pericardial closure, congenital/acquired heart defects, and blood vessels. Low rates of infection, complications and fibrotic adherence, and good hemodynamic behavior, have been reported. However, GA cytotoxicity and/or the incompletely shielded immunogenicity represent important limitations for the long-term performance of these pericardial substitutes and, hence, for the freedom from re-intervention in treated patients [10,11].

As we and other authors demonstrated [12,13,14,15,16], the decellularization of pericardium might be applied to produce biological scaffolds with improved immunocompatibility and unvaried biological and biomechanical properties with respect to native tissues. As an example, the clinical application of human acellular allogeneic pericardium as a patch during outflow tract reconstruction was effective in the midterm [17]. The demand for self-regenerating replacements is increasingly growing, but faces the raising paucity of human donated tissues. As a solution, biological substitutes might be unlimitedly obtained from other mammals. Nowadays, the clinical experience with heterologous acellular pericardium is still restricted, with few indications being available on the real long-term performance. Xenogeneic commercial products, as Tutopatch^TM^ [18], Cova^TM^ [19], Jason® [8], Integra® [20], and Osteokor pericardia [21], were released to treat several defects, requiring *dura mater* plastics, myringoplasty, digestive tissue reconstructions, bone and dental surgeries, cardiac corrections, or soft tissue augmentation to ameliorate facial and breast esthetics [22,23,24,25,26,27,28,29]. To prevent zoonosis risks, as Creutzfeldt-Jacobs disease, bovine tissues are derived from prion-free certified herd. These acellular membranes have been proved to behave as templates for guided tissue regeneration, by limiting inflammation and fibrosis and being resorbed in approximately 12–20 weeks. Almost 40 years ago, Tutogen medical (Alachua, FL, USA) developed the Tutoplast® technology to generate the bovine pericardial scaffolds Tutopatch^TM^ and Tutomesh^TM^. These devices were suitable in spinal *dura mater* reconstruction and in transcanal tympanoplasty [23,24,28]. PhotoFix®—a photo-fixed, decellularized bovine pericardial patch—revealed excellent performance in congenital cardiac surgery, similarly to autologous pericardium [30,31]. Decellularized equine pericardial patches, i.e., Matrix Patch™, were widely used in cardiosurgery: the outcomes of the five-year follow up have been announced to be disclosed soon [32,33].

A comparative assessment of acellular pericardia from different species is still incomplete, as opposed to the most largely implanted GA-preserved native tissues [34,35]. Such information is of paramount importance in developing effective therapeutic strategies targeted for each surgical indication. A comprehensive comparison of the most clinically applied pericardial replacements, i.e., bovine and porcine ones, has been performed after submitting native tissues to TRICOL decellularization [15,36,37,38,39,40], in order to verify whether decellularization exerts a differential effect on these tissues in terms of their histological, immunohistochemical, biochemical, and ultrastructural properties, as well as their denaturation, biomechanical, and cytocompatibility profiles.

## 2. Materials and Methods 

All reagents were supplied by Sigma-Aldrich (Saint Louis, MO, USA), unless otherwise specified.

### 2.1. Tissue Sample Dissection

Cadaveric cattle of the agri-food industry served as donors for the animal tissues used in this study. As a discard of this agri-food chain, no interventions were performed on animals and therefore, no ethical committee approval was required. 

Fresh native bovine and porcine pericardia (respectively, NBPs and NPPs) were collected from local abattoirs and transferred to the laboratory in phosphate-buffered saline (PBS) within 2 h. After thorough washing in PBS, pericardial specimens were isolated from bovine and porcine tissues and carefully cleaned from enveloping fat. 

Homogeneous areas of porcine and bovine pericardia (specifically for the bovine one, the left anterior region) were isolated from each animal (*n* = 6 per each species) and used for all performed analyses. In particular, region selection was operated on the basis of collagen uniformity of distribution, by using the technique of small-angle light scattering previously described by Sacks et al. [41].

### 2.2. TRICOL Decellularization Protocol

Decellularization was carried out according to TRICOL protocol [15,36,37,38,39,40]. Briefly, NBPs and NPPs were decellularized using protease inhibitors, alternated hypo/hypertonic solutions, and detergents, such as 0.1–1% (*w/v*) Triton X-100 and 10 mM sodium cholate. Residual nucleic acids were digested using 1500 U cm^−2^ of non-specific endonucleases (Benzonase™) at 37 °C for 48 h [36,37,38,39,40]. Obtained decellularized bovine and porcine pericardia, namely DBPs and DPPs, were maintained in antibiotics/antimycotic cold saline solution until further processing (100 U mL^−1^ penicillin, 100 mg mL^−1^ streptomycin and 250 µg mL^−1^ amphotericin B).

### 2.3. DNA Content

The total DNA content was quantified in NBPs, NPPs, DBPs and DPPs (*n* = 6 for each) by means of DNeasy Blood & Tissue Kit (Qiagen, Valencia, CA, USA) and measured at 260 nm with a NanoDrop 2000 spectrophotometer (Thermo Fisher Scientific, Waltham, MA, USA) after normalization per dry tissue weight. This latter was calculated indirectly by subtraction of average water content value (described later) from wet weight for each considered group.

### 2.4. Histological Evaluation

Histological analyses were performed on NBPs, NPPs, DBPs and DPPs following snap freezing in liquid nitrogen. Briefly, samples were fixed in 4% (*w/v*) paraformaldehyde (PFA) for 20 min at room temperature in darkness, maintained in 20% sucrose in PBS overnight at 4 °C and embedded in a 1:1 solution of 20% (*w/v*) sucrose and Optimum Cutting Temperature (OCT) compound (Tissue-Tek, Alphen Aan den Rijn, Netherlands). Tissue cryosections (7 µm) were stained with hematoxylin/eosin (H&E), Mallory trichrome (MT), Alcian Blue (AB), and Weigert-Van Gieson (VG), all supplied by Bioptica (Milan, Italy). Images were acquired with a Nikon Eclipse 50i light microscope equipped with a NIS-Elements D3.2 Software (Nikon Corporation, Shinagawa, Tokyo, Japan).

### 2.5. Immunofluorescence Staining

Indirect immunofluorescence was performed on 5–6 µm-thick cryosections of unfixed NBPs, NPPs, DBPs and DPPs to evaluate the effectiveness of TRICOL decellularization and analyze extracellular matrix (ECM) composition. The primary antibodies used were collagen I (1:100, C2456; Sigma), elastin (1:100, E4013; Sigma), collagen IV (1:100, ab6586; Abcam, Cambridge, UK), laminin (1:500, Z0097; Dako Cytomation, Glostrup, Denmark), and heparan sulfate (1:100, MAB1948P; Millipore, Darmstadt, Germany). Controls were incubated with 1% (*w/v*) bovine serum albumin instead of the primary antibodies. Rhodamine-conjugated, anti-mouse, anti-rat and anti-rabbit IgGs (1:100, Millipore) were applied as secondary antibodies to reveal primary antibody binding. Nuclei were counterstained with 4′,6-diamidino-2-phenylindole (DAPI). Images were acquired with an epifluorescence microscope Leica AF6000, connected to Leica DC300 digital camera and equipped with LAS AF Software (Leica Micro-systems, Wetzlar, Germany).

### 2.6. Scanning Electron Microscopy (SEM)

NBPs, NPPs, DBPs and DPPs were fixed and maintained in Karnovsky’s solution (8% (*w/v*) PFA and 10% (*v/v*) GA in 0.2 M cacodylate buffer) at 4 °C in the dark until assessment. Prior to SEM analysis, NBPs, NPPs, DBPs and DPPs were washed in physiologic solution (0.9% (*w/v*) sodium chloride) and dehydrated in a serial gradient of ethanol solutions (70%, 80%, 90% and 100% (*v/v*)) for 10 minutes each. Subsequently, samples were submitted to critical point-drying and coated with gold-palladium. Examination of the surface of the specimens was performed using JEOL JSM-6490 electron microscope (Jeol USA Inc., Peabody, MA, USA). Image acquisition was carried out using low vacuum mode (20 Kv) at two magnifications: 1.000x and 3.000x. 

### 2.7. Multiphoton Microscopy

Multiphoton-induced autofluorescence and second harmonic generation (SHG) were detected using a custom-built multimodal microscope, as recently described [42]. The chosen excitation wavelength was 800 nm in order to detect the SHG signal (400 nm) and the elastin autofluorescence signal (525 nm) on two different photodetectors (GaAsP PMT with 395/25 nm bandpass filter and GaAsP PMT with 525/40 nm bandpass filter, respectively). The average laser power used was 40 mW (30% of the maximum laser power), directly measured on the back-aperture of the objective by means of a Power Meter (Thorlabs, Inc, Newton, USA).

Images were acquired with a fixed resolution of 1024*1024 pixels and accumulation of 120 frames, with a pixel dwell time of 0.14 μs. For quantitative measurements, image acquisition was additionally performed in the format of RAW uncompressed files. Coherency, i.e., C, was calculated for collagen and elastin to verify the local dominant orientation in representative regions of interest (n = 9) using OrientationJ [43], an ImageJ plugin [44], as described by Rezakhaniha et al. [45]. The estimated parameter is comprised between 0 and 1, indicating respectively the absence (isotropy) and the presence (anisotropy) of dominant orientation. The quantification of the fiber waviness was realized at the zero-stress state as previously reported [8], by using the ImageJ plugin NeuronJ [46]. The straightness parameter P_s_ was defined as the ratio of the distance between two points of the collagen bundle (L0) and the corresponding length. A P_s_ value close to zero has to be considered indicative of a straight fiber.

### 2.8. Attenuated Total Reflectance FTIR analysis (ATR-FTIR)

The overall protein secondary structure of native and decellularized samples was studied using ATR-FTIR. Tissue punches of NBPs, NPPs, DBPs and DPPs were obtained using a biopsy puncher with a 6 mm diameter (Kai Europe, Solingen, Germany). In order to reduce the contribution of interfering water bands in the protein amide-I region, samples were equilibrated for 3-4 h in deuterium oxide. A Perkin-Elmer 100 FTIR spectrometer (Perkin-Elmer, Norwalk, CT, USA) was used to record infrared spectra. The spectrometer was equipped with a triglycine sulfate detector, and an ATR accessory with diamond/ZnSe crystal and pressure arm. Infrared spectra of the punches were recorded immediately after placing them on the ZnSe crystal of the ATR accessory, using the following parameters: 4 cm^−1^ resolution, 4 co-added interferograms, and 4000–900 cm^−1^ wavenumber range. From the acquired spectra, the 1700–1600 cm^−1^ region was selected. Second derivatives of the spectra were taken using a 13-point smoothing factor (Omnic software; Thermo-Nicolet, Madison, WI, USA) and normalized to resolve differences in peak intensities. Ratio of absorbance values of bands at 1650 and 1630 cm^−1^, respectively assigned to α-helical and ß-sheet structures, were calculated to quantify differences in amide-I band profile between native and decellularized groups using the following formula

R= Xi/Xj,(1)

where Xi and Xj represent the spectral absorbance values at 1630 or at 1650 cm^−1^, respectively.

### 2.9. Differential Scanning Calorimetry (DSC)

DSC measurements were performed using a Netzsch DSC 204F1 Phoenix instrument (Netzsch Gerätebau GmbH, Selb, Germany). Punches (6 mm in diameter) of NBPs, NPPs, DBPs and DPPs were weighed and transferred to aluminum pans, hermetically sealed and then analyzed. Samples were heated from 20 to 90 °C at a rate of + 10 °C min^−1^, with an empty pan used as a reference. Tissue protein denaturation could be visualized as an endothermic event in the DSC traces. Netzsch Proteus thermal analysis software (Netzsch Gerätebau GmbH) was used to obtain the onset temperature of protein denaturation (Tonset).

### 2.10. Biochemical Analyses for Hydroxyproline (HYP), Denatured HYP, Sulfated Glycosaminoglycans (sGAGs), Elastin and Water Quantification

The quantity of HYP, a collagen marker, was determined as described elsewhere [10]. NBPs, NPPs, DBPs and DPPs (*n* = 6 per each) were lyophilized and hydrolyzed with 6 N hydrochloric acid at 110 °C for 24 h, evaporated and dissolved in MilliQ water. Each sample and HYP standard (0.5–2.5 µg mL^−1^) were then oxidized by chloramine-T solution and incubated for 20 min at room temperature. Then, 19% perchloric acid was used to neutralize the oxidation reaction and the mixture allowed to stand for 5 min at room temperature. Finally, Ehrlich reagent (*p*-dimethylaminobezaldehyde solution) was added and each mixture was incubated again for 20 minutes at 60 °C and then cooled in tap water for 5 min. Absorbance of the resulting solution was read at 561 nm in a spectrophotometer (Bibby Scientific Limited, Stone, United Kingdom) and HYP concentration was calculated by interpolation from the standard curve and expressed as µg mg^−1^ of dry tissue.

The same groups described above for HYP quantification (*n* = 6) were also used to quantify denatured collagen, according to Bank et al. [47]. Moreover, autoclaved NBPs and NPPs were used as positive control. After α-chymotrypsin digestion, denatured collagen was quantified by detection of the HYP present in the supernatant, as described by Edwards & O’Brien [48]. Briefly, 10–15 mg of dry biomaterial per NBPs, NPPs, DBPs and DPPs was incubated with α-chymotrypsin solution (5 mg mL^−1^) in digestion buffer (0.1 M Tris and 2.5 mM calcium chloride; pH 7.8). Each supernatant was collected after centrifugation and hydrolyzed using 6 N hydrochloric acid (HCl) at 120 °C overnight. Every hydrolysate was then evaporated and treated with Ehrlich reagent, perchloric acid (Merck, Darmstadt, Germany) and chloramine-T. A calibration curve was obtained by preparation of serial concentrations of trans-4-hydroxy-L-proline (0–10 μg mL^−1^). Absorbance of each resulting solution was read at 570 nm in a spectrophotometer (Bibby Scientific Limited, Stone, United Kingdom) and the concentration of denatured HYP was calculated by interpolation from the standard curve and expressed as μg mg^−1^ of dry tissue.

sGAGs were quantified by using Blyscan sulfated glycosaminoglycan assay kit (B1000, Biocolor, Carrickfergus, United Kingdom). Samples were isolated from NBPs, NPPs, DBPs and DPPs (*n* = 6 per each) and digested with papain extraction solution at 65 °C overnight. Aliquots of each digested sample were mixed with 1,9-dimethyl-methyene blue dye to precipitate sGAGs-dye complex. A dissociation reagent was then used to release the bound dye into a solution and its absorbance was measured at 656 nm in a microplate reader (TECAN Infinite M2, Tecan Trading AG, Männedorf, Switzerland). sGAGs content was calculated based on standard curves generated from a reference solution composed of bovine tracheal chondroitin 4-sulfate (100 µg mL^−1^) and reported as µg mg^−1^ of dry tissue. The percentage change of sGAGs content from native to decellularized tissue was also estimated for each species.

Elastin quantification was performed with Fastin Elastin assay kit (F2000, Biocolor, Carrickfergus, United Kingdom). NBPs, NPPs, DBPs and DPPs (*n* = 6 per each) were treated with 0.25 M oxalic acid at 100 °C for two one-hour periods to extract α-elastin. Extracts were combined with Fastin dye reagent (5,10,15, 20-tetraphenyl-21H, 23H-porphine tetrasulfonate (TPPS)). Guanidine HCl, the dye dissociation reagent, and propan-1-ol were then added to release the dye into the solution, whose absorbance was measured at 513 nm in a microplate reader (TECAN Infinite M2). Standard curves were generated from reference solution of α-elastin prepared from bovine neck ligament (1 mg mL^−1^) in 0.25 M oxalic acid. Elastin content was reported as µg mg^−1^ of dry tissue.

Water content in NBPs, NPPs, DBPs and DPPs (*n* = 6 for each type) was calculated by the difference in wet and dry weight and expressed as percentage of the wet weight. Before weighing, wet samples were gently blotted on a filter paper and equilibrated for two minutes. For dry weight, specimens were freeze-dried (Modulyo Freeze Dryer, Edwards, Crawley, UK) overnight and then weighed.

### 2.11. Uniaxial Tensile Testing

Fresh (native) and decellularized pericardial samples (*n* = 6 for each type and species, 10 mm × 5 mm) were isolated by cutting parallel to the collagen fiber alignment using a light box and two polarized light filters. 

Tissue thickness was measured while using a digital gage (accuracy: ± 0.1 mm; Mitutoyo, Andover, UK). As previously described by Korossis et al. [49], specimens were subjected to low strain rate uniaxial tensile loading to failure testing by means of an Instron® tensile machine (Instron 3365; Instron, Bucks, UK) that was equipped with a temperature-controlled bath (PBS, 37 °C). The tissue samples were preconditioned for 50 cycles at a rate of 20 mm min^−1^, before being consecutively stretched to failure at the same rate. During testing, load (F, Newton) and extension (Δl, mm) were recorded and converted to engineering stress (σ, MPa) and strain (ε, adimensional). The stress-strain curve of each sample was plotted and four parameters were calculated: elastic phase modulus (MPa), collagen phase modulus (MPa), failure strain (%), and ultimate tensile strength (MPa; UTS) [49]. Finally, the calculated biomechanical parameters were averaged over the number of samples in each group.

### 2.12. In Vitro Cytotoxicity Assays

In vitro cytotoxicity of decellularized pericardial scaffolds was assessed quantitatively and qualitatively by means of contact assay, as described into the dedicated part 5 of ISO 10993 concerning the biological evaluation of medical devices [50]. Human BM-MSCs at passage 7 and HUVECs at passage 6 (all from PromoCell, Heidelberg, Germany) were used to perform these analyses. 

DBPs and DPPs were cut with biopsy punchers (8 mm diameter; Kai industries) and permanently fixed to a six-well culture plate (Corning Inc., Lowell, MA, USA) using sterile adhesive strips (3M, Maplewood, MN, USA). Prior to cell seeding, a 24 h-treatment with an antibiotics/antimycotic cocktail was performed to provide scaffold disinfection, as previously described by Fidalgo et al. [40]. Samples (*n* = 3 per each species) were then washed three times with PBS and kept overnight in basal endothelial or alpha MEM medium at 37 °C until seeding with HUVECs or hBM-MSCs, respectively. Both cell types were plated at a density of 1.5 × 10^5^ cells per well with 2 ml of culture medium and maintained in contact with the samples for 3 days. Wells with no scaffold samples or cyanoacrylate glue (3M Italia, Pioltello, Italy) were seeded at the same cell density and served as positive and negative controls, respectively, at each time point. 

For qualitative contact assay, cells were fixed with 2% (*w/v*) PFA and stained with Giemsa staining (Merck, Darmstadt, Germany) to visualize cell morphology and distribution under light microscopy. Images acquisition was realized with a Nikon Eclipse 50i light microscope (Nikon). 

In order to evaluate possible proliferation changes, an MTS assay (CellTiter 96®AQueous One Solution Cell Proliferation Assay, Promega, Madison, WI, USA) was performed. Cell proliferation was assessed at 24 and 72 h. The reduction of MTS tetrazolium compound by viable, proliferating cells into a formazan-dye-product was quantified by measuring the absorbance at 490 nm in a plate reader spectrophotometer (FLUOstar Omega, BMG LABTECH, Ortenberg, Germany).

A cytotoxicity assay was realized by measuring lactate dehydrogenase (LDH) released in the culture media after cell lysis by means of a colorimetric assay based on the conversion of tetrazolium salt in formazan (Pierce LDH Cytotoxicity Assay Kit, Thermo Fisher Scientific). Absorbance was acquired at 490 nm using a plate reader spectrophotometer (FLUOstar Omega) and elaborated as percentage of cytotoxicity, as previously described by Cebotari et al. [51].

### 2.13. Statistical Analysis

All variables were expressed as mean ± standard deviation (SD). Normality was checked with Q-Q plots and presence of outliers identified by the ROUT method with a Q level of 1% (maximum permitted false discovery rate) [52]. Group comparisons were conducted with one-way ANOVA not assuming equal standard deviation between groups (Welch test). Pairwise multiple comparisons were corrected for false discovery rate with the two-stage linear step-up procedure of Benjamini, Krieger and Yekutieli [53] applied at each experiment type. Significance was set at *p* < 0.05. GraphPad Prism version 8.2.0 for Windows (GraphPad Software, San Diego, California, USA) was used for statistical analysis.

## 3. Results

### 3.1. Cell Removal from Bovine and Porcine Pericardia after TRICOL Decellularization

**Figure 1:**
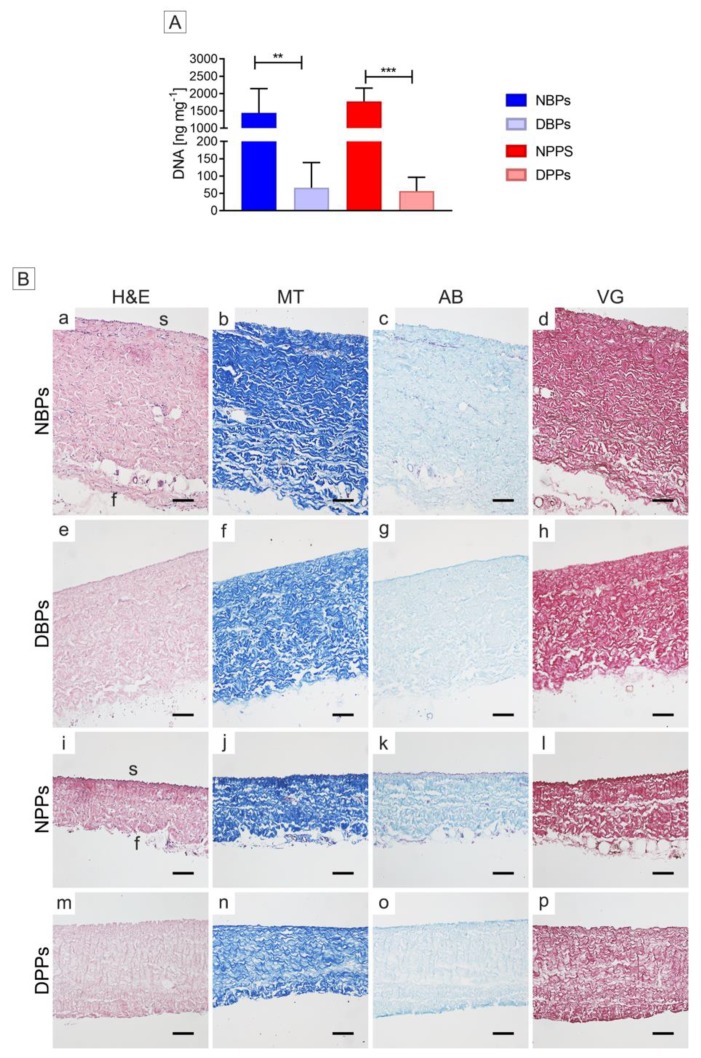
Decellularization yield of bovine and porcine pericardia after TRICOL procedure. (**A**) DNA content before and after decellularization. The data are presented as mean ± standard deviation (SD). DNA was significantly reduced in decellularized scaffolds when compared to native ones. **p < 0.01; ***p < 0.001. (**B**) Histological evaluation before and after decellularization. Intact extracellular matrix (ECM) and no nuclear material were revealed in decellularized bovine (e) and porcine (m) pericardia with respect to their native counterparts (a and i) (Haematoxylin and eosin (H&E)). Collagen organization was intact after decellularization (f for bovine and n for porcine), as in the native corresponding samples (b and j) (Masson trichrome (MT)). A light discoloration was observed for Alcian blue (AB) in decellularized bovine (g) and porcine (o) pericardia with respect to their native counterparts (c and k). No changes in the elastic fibers were detected between the bovine and porcine ECM of native (d and l, respectively) and decellularized (h and p, respectively) pericardia (Van Gieson (VG)). Magnification bars: 100 µm. s= *serosa*, f= *fibrosa*. NBP = Native Bovine Pericardium, DBP = Decellularized Bovine Pericardium, NPP = Native Porcine Pericardium, and DPP = Decellularized Porcine Pericardium.

After TRICOL treatment, the DNA amount was significantly reduced by 97.48% and 98.43% in bovine and porcine TRICOL samples (DBPs and DPPs) with respect to the original native tissues (namely NBPs and NPPs; *p* = 0.0046 and *p* = 0.0001) (Figure 1A and Table S1). 

### 3.2. Gross Structure of Decellularized Bovine and Porcine Pericardia

TRICOL decellularization maintained the general ECM organization of parietal pericardium with distinct *serosa* and *fibrosa* layers in both species. Original cells (Figure 1B a–d for NBPs and i–l for NPPs) were efficiently removed by TRICOL protocol (Figure 1B e–h for DBPs and m-p for DPPs). Collagen bundles of DBPs and DPPs presented the characteristic wavy pattern observed in NBPs and NPPs (Figure 1B b and j). After decellularization, the *fibrosa* outer and looser connective tissue scaffolds acquired a compact appearance in both species (Figure 1B f for DBP and n for DPP). DBPs and DPPs exhibited general discoloration for sulfated mucopolysaccharides (Figure 1B g and o, respectively), in respect to the native condition (Figure 1B c for NBPs and k for NPPs).

With reference to NBPs and NPPs (Figure 1B d and l, respectively), the TRICOL procedure did not affect elastin pattern around the collagen bundles in the whole thickness and in the blood vessels’ arterial wall of treated pericardial scaffolds of both species (Figure 1B h for DBPs and p for DPPs).

### 3.3. ECM Architecture (Basal Lamina Included) of Bovine and Porcine Pericardia after TRICOL Decellularization

**Figure 2:**
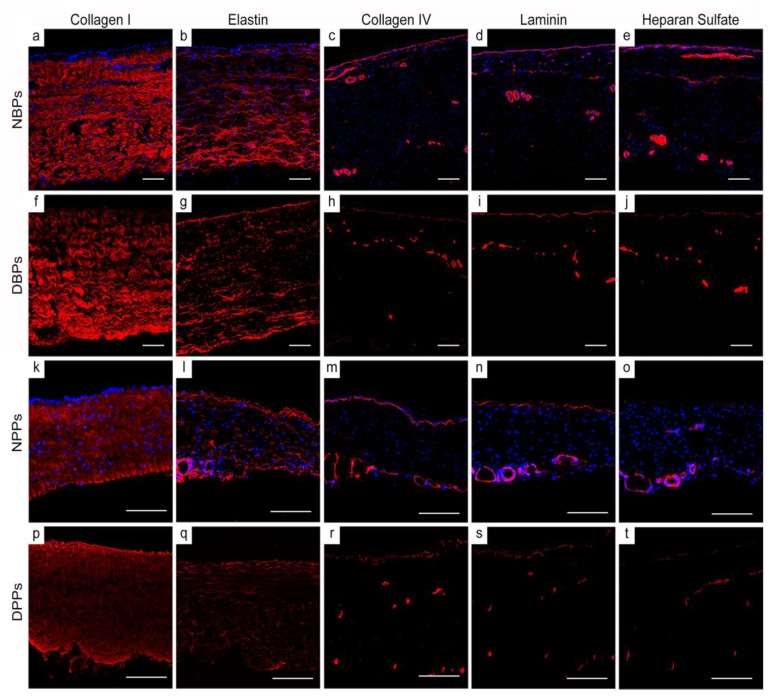
Extracellular matrix (ECM) properties of bovine and porcine pericardia before and after TRICOL decellularization. The general histoarchitecture of collagen and elastin was preserved after TRICOL decellularization (red; f and g for bovine ECM; p and q for porcine ECM) with respect to the native condition (red; a and b for bovine ECM; k and l for porcine ECM). The basal lamina elements, i.e., collagen IV, laminin and heparan sulfate, were not removed in TRICOL-treated tissues (red; h-j for bovine ECM and r-t for porcine ECM) as compared to native samples (red; c-e for bovine ECM and m-o for porcine ECM). Nuclei, clearly visible with DAPI in native tissues (blue; a–e for bovine tissues; k–o for porcine tissues), were absent after decellularization (f–j for bovine scaffolds; p–t for porcine scaffolds). Magnification bars: 100 µm. NBP = Native Bovine Pericardium, DBP = Decellularized Bovine Pericardium, NPP = Native Porcine Pericardium, and DPP = Decellularized Porcine Pericardium.

Wavy collagen I fibers were observed by immunofluorescence in both native (Figure 2 a for NBPs and k for NPPs) and decellularized (Figure 2 f for DBPs and p for DPPs) tissues. The elastin network was well-preserved in TRICOL-treated pericardia (Figure 2 g for DBPs and q for DPPs) when compared to the native counterparts (Figure 2 b for NBPs and l for NPPs). TRICOL did not affect the distribution of collagen IV, laminin and heparan sulfate, i.e., the main elements composing the basal lamina (Figure 2 c–e for NBPs, h–j for DBPs, m–o for NPPs, and r–t for DPPs). DAPI staining reconfirmed the removal of nuclear material (Figure 2 f–j for DBPs and p–t for DPPs).

**Figure 3:**
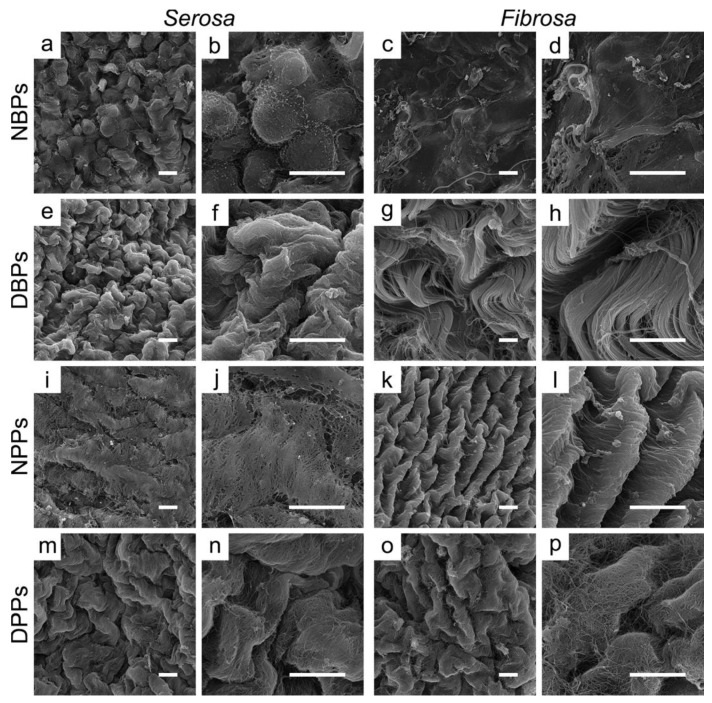
Ultrastructural properties of bovine and porcine pericardia before and after TRICOL decellularization. The mesothelial cell lining of the *serosa* side of bovine (a and b) and porcine (i and j) pericardia was removed after decellularization (e and f for bovine; m and n for porcine). The collagen bundles were loosened in the native bovine *fibrosa* (c and d), becoming more organized after decellularization (g and h), while they were crimped for the porcine counterparts (k and l for native; o and p for decellularized). Magnification bars: 10 µm. NBP = Native Bovine Pericardium, DBP = Decellularized Bovine Pericardium, NPP = Native Porcine Pericardium, and DPP = Decellularized Porcine Pericardium.

At SEM, mesothelial cells, cobblestone-like in the native *serosa* of NBPs (Figure 3 a and b) and NPPs (Figure 3 i and j), were not revealed in DBPs and DPPs (Figure 3 e and f, and m and n). Wavy collagen bundles of sub-mesothelial connective tissue were evident (Figure 3 e for DBPs and m for DPPs). In the *fibrosa*, collagen bundles were looser in NBPs and DBPs (Figure 3 c and d, g and h, respectively) than in NPPs and DPPs (Figure 3 k and l, o and p, respectively), and wider than in the *serosa* (Figure 3 e and m, respectively). At higher magnification, collagen and elastin organization appeared unaltered by TRICOL decellularization (Figure 3 f and h for DBPs and n and p for DPPs).

**Figure 4:**
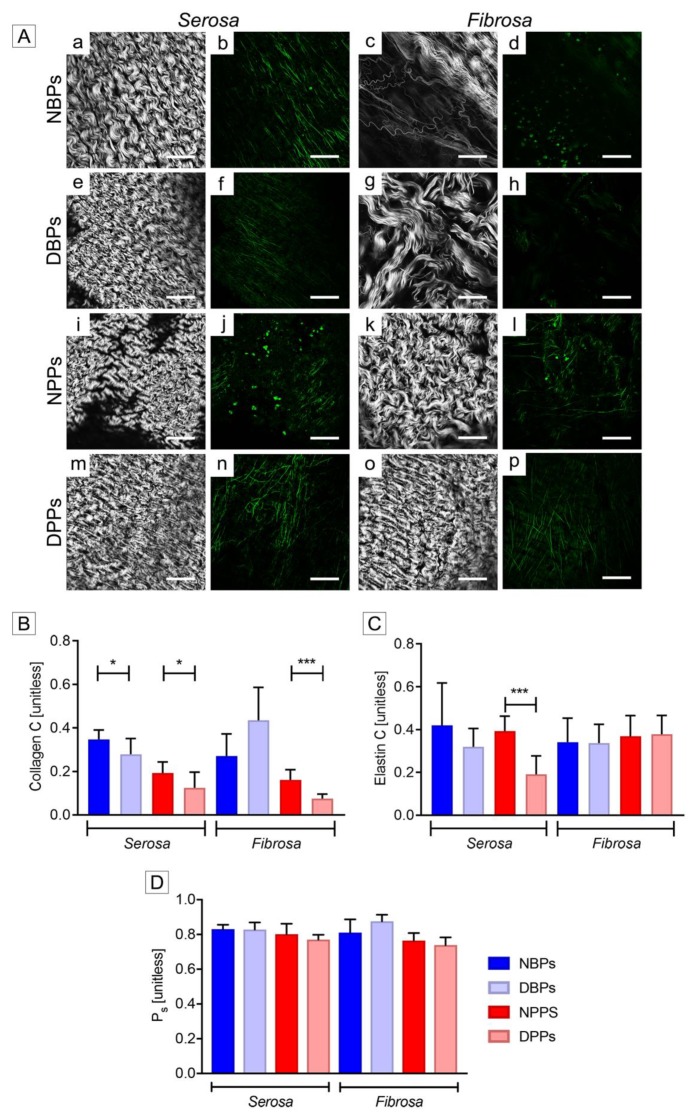
Extracellular matrix (ECM) pattern of bovine and porcine pericardia before and after TRICOL decellularization. (**A**) Second harmonic generation and autofluorescence. In the decellularized serosa (e and f for bovine; m and n for porcine), collagen and elastin maintained the tight organization of the native condition (a and b for bovine; i and j for porcine). Bovine acellular fibrosa (g and h) showed an unaltered collagen loosen distribution with respect to native tissue (c and d). A more crimped and wavy appearance was shown in the decellularized porcine counterpart (k and l for native and o and p for decellularized). Magnification bars: 100 µm. (**B**) Collagen coherency (C). C of collagen bundles decreased significantly for decellularized serosa of both species and decellularized porcine fibrosa with respect to native tissues. (**C**) Elastin coherency (C). C of elastin fibers was significantly higher in native porcine serosa samples than in decellularized ones. (**D**) Straightness parameter (P_s_) of collagen bundles. No significant modifications were revealed after decellularization for the P_s_ of collagen, apart from bovine fibrosa. For **B**, **C** and **D**, data are presented as mean ± standard deviation (SD). *p < 0.05; ***p < 0.001. NBP= Native Bovine Pericardium, DBP= Decellularized Bovine Pericardium, NPP= Native Porcine Pericardium, DPP= Decellularized Porcine Pericardium, C= coherency, and P_s_= straightness parameter.

SHG analyses confirmed the crimped and layer-specific organization of collagen in both native and decellularized pericardia (*serosa:* Figure 4A a for NBPs, e for DBPs, i for NPPs and m for DPPs; *fibrosa*: Figure 4A c for NBPs, g for DBPs, k for NPPs, o for DPPs). In decellularized *serosa*, collagen reorganization contributed to a significant C decrease (Figure 4B; for DBPs, *p* = 0.0299; for DPPs, *p* = 0.0364). In *fibrosa* layer, this parameter was significantly augmented after decellularization only for porcine tissues (*p* = 0.0004). TRICOL decellularization did not modify the optical properties of elastin (Figure 4A f and h for DBPs, n and p for DPPs). Elastic fibers appeared running parallel to collagen in NBPs (Figure 4A b and d) and in DBPs (Figure 4A f and h), while formed a network in NPPs and DPPs (Figure 4A j and l and n and p, respectively) with a significant C reduction in the porcine *serosa* after decellularization (Figure 4C; for DPPs, *p* = 0.0009). The two-photon excitation at 800 nm enabled detecting cells within native bovine (Figure 4A b and d) and porcine pericardia (Figure 4A j and l). No cells or nuclear material could be detected in DBPs and DPPs (Figure 4A f and h for DBPs and n and p for DPPs), by confirming previous microscopical analyses. Generally, cell extraction induced a tighter organization of the ECM, with significant P_s_ increase only for bovine *fibrosa* (*p* = 0.0472) (Figure 4D).

### 3.4. Differences of Bovine and Porcine Pericardia after TRICOL Decellularization

**Figure 5:**
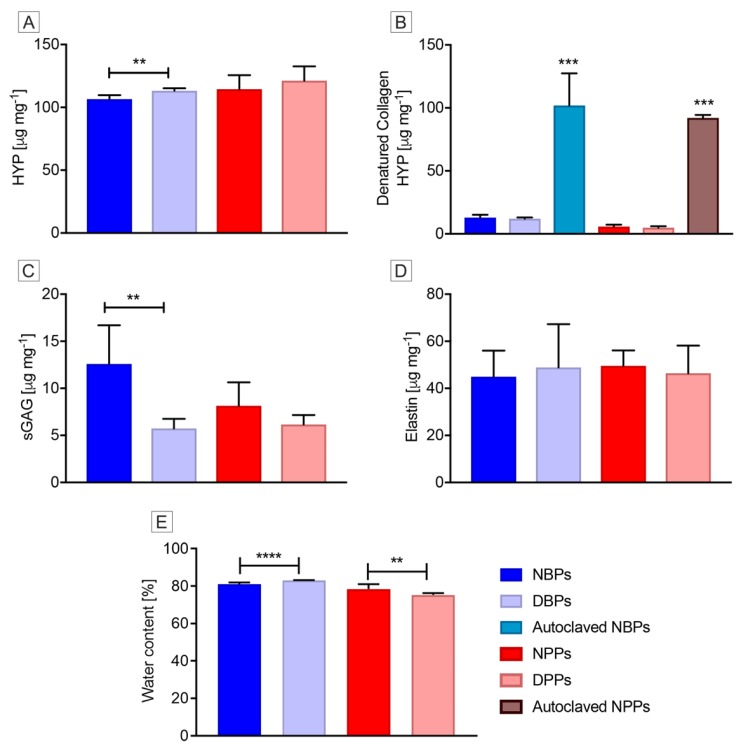
Quantitative biochemical profile of bovine and porcine pericardia before and after TRICOL decellularization. (**A**) Hydroxyproline (HYP) content. Estimated amount of HYP appeared superior in decellularized bovine tissues especially. (**B**) Denatured HYP. No denaturation was observed after decellularization in both species, as opposite to positive controls (autoclaved tissues). (**C**) Sulfated glycosaminoglycans (sGAGs) content. TRICOL decellularization decreased sGAGs, significantly in bovine pericardium. (**D**) Elastin content. No significant differences were revealed for elastin content. (**E**) Hydration level. Water content modified significantly after decellularization: it increased for bovine pericardium, while decreased for porcine one. Data are presented as mean ± standard deviation (SD). *p <0.05; **p <0.01; ***p <0.001; ****p < 0.0001. NBP= Native Bovine Pericardium, DBP= Decellularized Bovine Pericardium, NPP= Native Porcine Pericardium, and DPP= Decellularized Porcine Pericardium.

After TRICOL, the HYP content was determined to be higher with a significant difference in the case of bovine tissues (*p* = 0.0063) (Figure 5A and Table S1). This apparent increase of HYP content is due to the loss of cells and soluble proteins, which outweighs collagen quantity variation. 

No significant difference was detected for denatured collagen concentrations in native and decellularized pericardia of both species (Figure 5B and Table S1), conversely to autoclaved tissues. 

The TRICOL procedure resulted in a sGAGs loss in treated pericardia (Figure 5C and Table S1). The percentage decrease in sGAGs content for bovine and porcine tissues was 54.33% and 24.54%, respectively, with a significant difference for the bovine pericardium (*p* = 0.0085).

The elastin content was not affected by decellularization for both species’ samples (Figure 5D).

Regarding the water content, the hydration level significantly changed after decellularization: it increased in bovine scaffolds (*p* < 0.0001), while it decreased in porcine ones (*p* = 0.0031) (Figure 5E and Table S1).

### 3.5. Overall Protein Sequence and Denaturation Profile of Bovine and Porcine Pericardia after TRICOL Decellularization

**Figure 6:**
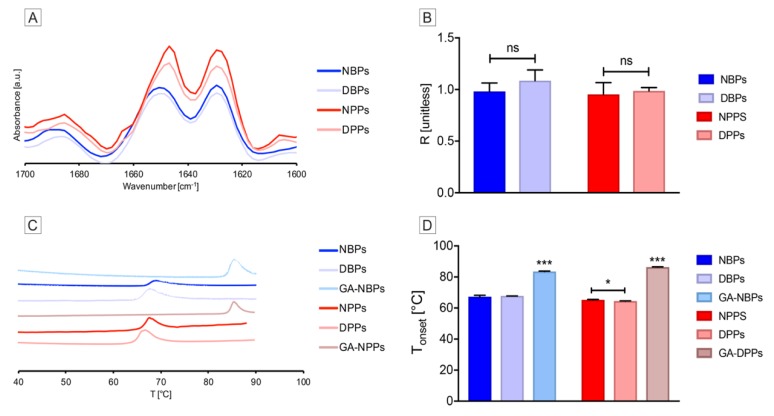
Denaturation profiles of bovine and porcine pericardia before and after TRICOL decellularization. (**A**) Second derivatives of the recorded spectra between 1600 and 1700 cm^−1^. No modifications were induced by TRICOL decellularization on the secondary structure, i.e., α-helical structures (1650 cm^−1^) and ß-sheets (1630 cm^−1^). (**B**) R ratio. R was not significantly modified by TRICOL decellularization in both species’ tissues. (**C**) Differential scanning calorimetry (DSC) thermograms. Similar profiles were observed when decellularized and native pericardia of both species were compared. Glutaraldehyde treatment sensibly modified tissue thermograms. (**D**) The onset temperature (T_onset_) of protein denaturation. Denaturation occurred at decreased temperature with respect to native tissues only for porcine pericardium. Glutaraldehyde treatment caused a significant increase of the denaturation temperature for both species with respect to all other groups considered. Data are presented as mean ± standard deviation (SD). *p <0.05, ***p <0.001. NBP= Native Bovine Pericardium, DBP= Decellularized Bovine Pericardium, NPP= Native Porcine Pericardium, DPP= Decellularized Porcine Pericardium, and GA= Glutaraldehyde.

ATR-FTIR analysis—essential for studying the effect of decellularization on the overall protein secondary structure—confirmed the existence of α-helical and β-sheet structures (respectively, 1650 and 1630 cm^−1^) in the spectral fingerprint of all native and decellularized tissues (Figure 6A). The R ratio of the bands at 1650 and 1630 cm^−1^ was used as a parameter of comparison. The calculated R values were 0.96 for NBPs and DBPs, 0.95 for NPPs, and 0.98 for DPPs (Figure 6B), with no significant differences, indicating no alterations of the protein secondary structure by TRICOL decellularization.

DSC was used to provide a thermal tissue fingerprint to assess protein stability before and after decellularization (Figure 6C). T_onset_ was compared among the native, decellularized, and GA-treated pericardia of each species, and was determined to be 66.7 ± 0.9, 67.77 ± 0.07, and 83.55 ± 0.25 °C for the bovine Group, and 65.4 ± 0.25, 64.45 ± 0.15, and 86.45 ± 0.17 °C for the porcine One (Figure 6D). A slight variation of T_onset_ was found to be significant for DPPs with respect to NPPs (*p* = 0.0146), but not between NBPs and DBPs (*p* = 0.4838). 

Taken together, these results suggested that the TRICOL decellularization procedure did not induce denaturation, thus corroborating the biochemical outcomes.

### 3.6. Biomechanical Properties of Bovine and Porcine Pericardia after TRICOL Decellularization

**Figure 7:**
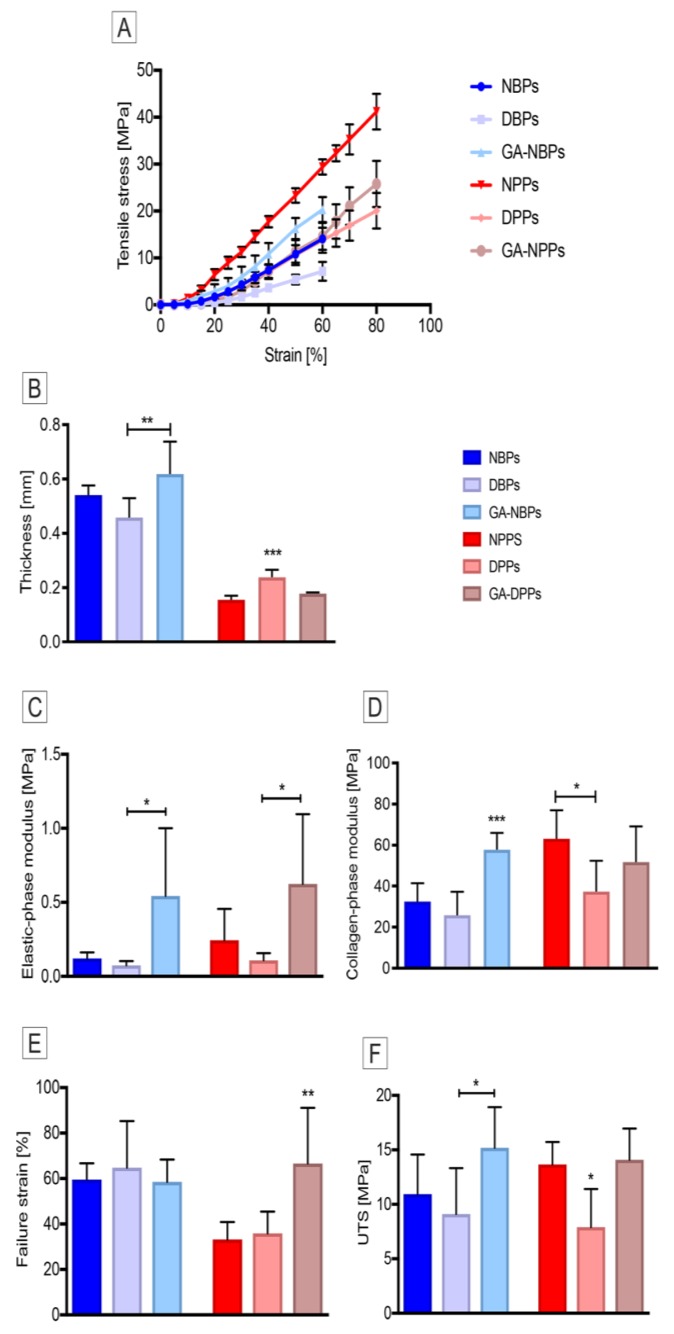
Biomechanical properties of bovine and porcine pericardia before and after TRICOL decellularization. (**A**) Stress-strain curves. Typical J-shaped curves of soft tissues were appreciated for all tested pericardial samples. (**B**) Thickness. As compared to native tissues, TRICOL decellularization induced no significant changes in bovine pericardium thickness, while varied porcine one. Glutaraldehyde (GA) increased significantly the thickness of bovine pericardium, but decreased that of porcine scaffold. (**C**) Elastic-phase modulus. TRICOL decellularization had no effects on elastic-phase modulus. (**D**) Collagen phase modulus. After TRICOL decellularization, both bovine and porcine pericardial scaffolds showed a decrease in collagen phase modulus, although significant only for the second species. GA significantly increased collagen phase modulus of bovine pericardium. (**E**) Failure strain. No significant failure strain modifications were reported after TRICOL decellularization, while a significant rise was observed after GA treatment in porcine tissues. (**F**) Ultimate tensile strength (UTS). TRICOL decellularization tended to reduce ultimate tensile strength of both species’ tissues, without any statistical significance. The data are presented as mean ± standard deviation (SD). *p< 0.005, **p< 0.01, ***p< 0.001. NBP= Native Bovine Pericardium, DBP= Decellularized Bovine Pericardium, NPP= Native Porcine Pericardium, DPP= Decellularized Porcine Pericardium, and GA= Glutaraldehyde.

During low-strain rate uniaxial loading to failure, pericardial samples exhibited a J-shaped stress-strain curve (Figure 7A), as typically observed for all soft tissues [54]. After TRICOL decellularization, thickness decreased for bovine pericardium (0.46 ± 0.06 mm for DBPs with respect to 0.54 ± 0.04 mm for NBPs; *p* = 0.0614), but significantly increased for porcine pericardium (0.24 ± 0.02 mm for DPPs versus 0.15 ± 0.01 mm for NPPs, *p* = 0.0002) (Figure 7B). A trend towards lower values in elastic- and collagen-phase moduli was evident for bovine and porcine pericardia after TRICOL decellularization (Figure 7C and D), with significant reductions only for the second parameter in DPPs in comparison to NPPs (respectively, 37.33 ± 11.12 MPa and 59.07 ± 8.02 MPa, *p* = 0.0117; Figure 7D). Similar behaviors were also observed for failure strain (Figure 7E). DBPs and DPPs exhibited higher moduli than the corresponding native ones, indicating the acquisition of larger deformability. Additionally, no significant differences were found as to the UTS values of decellularized pericardia with respect to native specimens (Figure 7F). These observations suggested that decellularized bovine and porcine pericardia were both slightly compliant when compared to their native counterparts.

A non-significant slope increase was observed in the elastic-phase modulus of GA-treated bovine (0.24 ± 0.83 MPa, *p* = 0.1330) and porcine (0.58 ± 0.77 MPa, *p* = 0.0702) samples as compared to their decellularized counterparts (0.07 ± 0.02 MPa and 0.11 ± 0.04 MPa), indicating a loss of extensibility induced by GA (Figure 7C). A similar trend was detected for the failure strain of these GA-treated tissues when compared to native and decellularized samples, though this was significant only for the porcine Group (Figure 7E, *p* = 0.0356 and *p* = 0.0460, respectively). In addition, the collagen-phase modulus tended to increase due to GA treatment for both species (Figure 7D), being the difference significant only for bovine pericardium compared to native (*p* = 0.0009) and decellularized (*p* = 0.0013) ones.

### 3.7. Bioactive Properties of Bovine and Porcine Pericardia after TRICOL Decellularization

**Figure 8:**
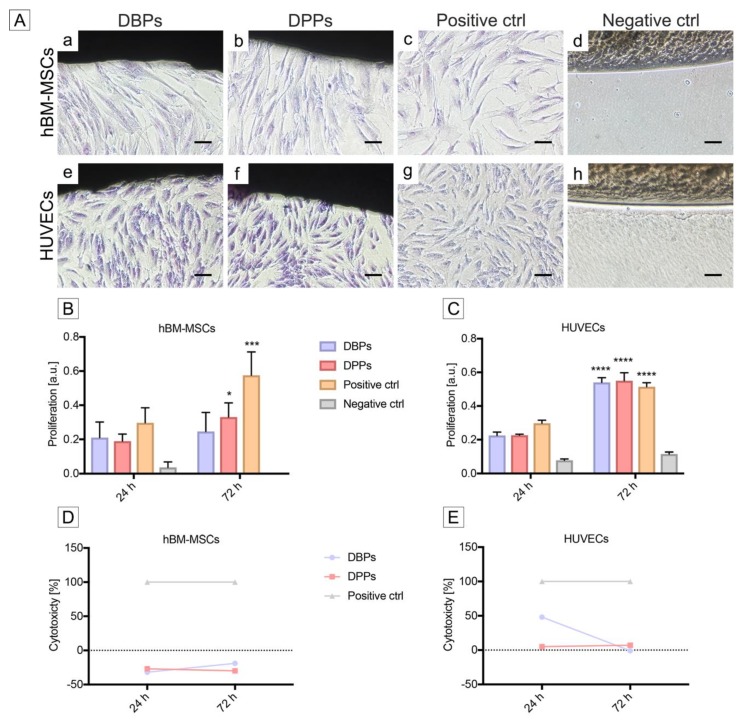
Bioactivity of bovine and porcine pericardia after TRICOL decellularization. (**A**) Contact assay with human bone marrow mesenchymal stem cells (hBM-MSCs) and umbilical vein endothelial cells (HUVECs). Both cell types displayed typical morphology, respectively spindle-shape and cobblestone-like, when cultured in contact with bovine and porcine decellularized scaffolds (a and b, as well as e and f), similarly to seeded alone (positive controls: c and g). In the cytotoxic glue environment (negative control), no cell growth was evident (d and h). Scale bar: 100 µm. (**B**) Proliferation assay for hBM-MSCs. When compared to positive controls (culture plastic support), no proliferation differences were observed at 24 h, while increases were revealed in respect to both decellularized scaffolds and between porcine and bovine ones. The data are presented as mean ± standard deviation (SD). * *p* < 0.5, *** *p* < 0.001, **** *p* < 0.0001. (**C**) Proliferation assay for HUVECs. Differences in proliferation were not evident at 24 h, but at 72 h among all groups considered. Data are presented as mean ± SD. Significance differences are shown inside the same group with respect to time. * *p* < 0.5, *** *p* < 0.001, **** *p* < 0.0001. (**D**) Lactate dehydrogenase (LDH) cytotoxicity assay for hBM-MSCs. The direct contact with decellularized bovine and porcine pericardial scaffolds did not induce hBM-MSCs cytotoxicity either at 24 or 72 h. (**E**) LDH cytotoxicity assay for HUVECs. Cytotoxicity of HUVECs was observed high at 24 h and decreased near 0 at 72 h after seeding onto decellularized bovine and porcine pericardial scaffolds. Data are presented as mean ± SD. DBP= Decellularized Bovine Pericardium, DPP= Decellularized Porcine Pericardium, hBM-MSCs= human bone marrow-mesenchymal stem cells, and HUVECs= human umbilical vein endothelial cells..

During cytocompatibility assays [50], the seeded hBM-MSCs and HUVECs presented at 72 h sub-confluence pattern and regular morphology, i.e., respectively, spindle-shape fibroblast-like and cobblestone-like phenotypes, as well as sustained proliferation (mitotic phase), and definite polarization towards DBPs and DPPs (Figure 8A a and e for DBPs, b and f for DPPs and c and g for positive controls, concerning hBM-MSCs and HUVECs, respectively). These observations were equivalent to the cytotoxicity grade “0” of ISO 10993-5 [50]. The glue (negative control) prevented cell adhesion, thus confirming its high cytotoxic effect (Figure 8A d for hBM-MSCs and h for HUVECs).

The proliferation activity of hBM-MSCs in contact with DBPs did not vary significantly over time, while it increased for DPPs and positive control (Figure 8B). Considering HUVECs, the increase was significant also for DBPs (Figure 8C). In addition, a significantly rising proliferation was revealed in all groups with respect to negative controls, independently on time evaluation and cell type, except at 24 h for DPPs seeded with hBM-MSCs (data not shown). No significant differences were observed in cell proliferation with respect to the positive controls at 24 h for DBPs that were seeded with hBM-MSCs and at 72 h for the same scaffolds seeded with HUVECs (data not shown).

The cytotoxicity values for hBM-MSCs remained below zero for all time points after contact with both DBPs and DPPs (Figure 8D). In the case of HUVECs, the cytotoxicity was recorded higher at 24 h for DBPs, decreasing rapidly to zero at 72 h, while it remained negligible for DPPs (Figure 8E). The positive control (glue) induced 100% cytotoxicity (Figure 8 D and E). In agreement with ISO 10993-5 [50], bioactivity was observed unaltered after decellularization for both species’ tissues.

### 3.8. Differences of TRICOL Decellularized Bovine and Porcine Pericardia 

A schematic comparison of all quantitative parameters for bovine and porcine pericardia, before and after TRICOL decellularization, and the evaluation of their statistical significance is provided in Table 1. 

In the comparative analysis between native bovine and porcine tissues, the properties of the ECM architecture and structure appeared to be more conserved among different species. Differently, ECM biochemical composition—especially sGAGs content and hydration level—, T_onset_ and biomechanical behavior, including thickness, collagen phase modulus, and failure strain, were significantly differing between the native samples. 

With respect to the assessment of the decellularization effects on native tissues, the bovine scaffolds seemed to have undergone more significant changes, particularly in terms of collagen properties, as coherency and amount—even if this could be an artifact due to the loss of cells and consequent weight/elements proportion variation, as pointed before; no dissimilarities were instead revealed for collagen phase modulus. Besides collagen quantity, also sGAGs and water ones significantly modified after TRICOL decellularization of bovine pericardia. The hydration level also changed for porcine tissues, but specific modifications for this species were only observed for coherencies of serosal elastin and fibrosal collagen, thickness, and collagen phase modulus.

By comparing DBPs and DPPs, the original significant differences that were observed for NBPs and NPPs in serosal and fibrosal collagen C, denatured collagen water content, thickness, collagen phase modulus, and failure strain were maintained or slightly reduced. With respect to NBPs and NPPs, the comparison between their decellularized scaffolds revealed that TRICOL treatment only modified fibrosal P_s_ and T_onset_, while the initial significant difference in collagen phase modulus between the two species’ scaffolds was nullified. Moreover, no significant variations were revealed in the ECM bioactive properties between DBPs and DPPs after cell contact challenge with both hBM-MSCs and HUVECs.

## 4. Discussion

The use of biomimetic materials, such as autologous and allogeneic pericardia, seems to be a suitable response to the dramatically increasing clinical need for functional bioreplacements. In fact, pericardial tissues are characterized by an ECM scaffolding, which is able to guarantee pliability, conformability, and resistance to compression, but also to promote healing by repopulating cells. Moreover, they can be easily sutured by providing sufficient strength [55] and they are not immediately resorbable, thus constituting an efficient barrier. All of these features render them excellent biological membranes for guided tissue regeneration.

In the therapeutic perspective of auto- or allotransplantation, patients’ comorbidities and donors’ scarcity represent critical limitations. Xenogeneic pericardia demonstrated possessing similar characteristics with respect to human ones; however GA-treatment has to be applied before clinical use to prevent catastrophic immune responses and, hence, graft failure [10,11]. 

Decellularized xenogeneic pericardia of different species [18,19,21,30], preferentially bovine, have been demonstrated to offer similar advantages and overcome the restrictions of human and GA-treated heterologous tissues in reconstructive or substitutive surgery [22,23,24,25,26,27,28,29]. Yet, no comparative studies have thoroughly investigated the decellularization effects on tissues from different species. 

To our knowledge, this work represents the first attempt to assess any tissue specificity in a comparison between bovine and porcine pericardia before and after detergent decellularization, i.e., TRICOL procedure. Previously, we reported its efficacy in the decellularization of porcine and human cardiovascular tissues [15,36,37,38,39]. This study demonstrates that TRICOL is effective in cell extraction from both bovine and porcine pericardia, without any adverse effects on both species’ ECMs. The original difference in tissue thickness between the two species is maintained. The main fibrous components, as collagen I and elastin, as well as the elements of the basal lamina, are preserved with no protein denaturation or degradation. 

Decellularization induces a decrease in the original amount of sulfated GAGs, as observed earlier by our and other groups [12,15,55,56,57]. Although the loss of these weakly ECM-linked molecules might be supposed as beneficial to facilitate diffusion during decellularization and repopulation [56], its effects should be closely evaluated in a clinical application perspective. In native porcine pericardium, the major chondroitin sulfate isomer is dermatan sulfate and TRICOL decellularization reduces GAGs, without substantially modifying the distribution of their isomers [15]. Although the eventually differential GAGs extraction from the two species’ tissues was beyond the scope of this research, it might be hypothesized that the removal of these molecules might have possible consequences for several biological and pathophysiological processes [58,59,60,61].

sGAGs have been associated to complement modulation in inflammation and immune response [62,63]. Thus, their reduction/absence in biomaterials might be considered as advantageous in cardiovascular settings to prevent calcification and/or immune responses. Nonetheless, their presence could be profitable for dental and bone surgical applications aiming at inducing an accelerated mineralization process (Table 2). 

sGAGs are likewise involved in the maintenance of the homeostasis of tissue hydration. In native conditions, bovine pericardial tissues possess a higher water content than porcine counterparts. This difference increases after decellularization, apparently without a direct link to the more prominent sGAGs loss that was observed in the bovine scaffolds. 

The reduction of sGAGs has been described as being detrimental on the biomechanical behavior of connective tissues [64,65]; however, TRICOL decellularized scaffolds likely revealed only a tendency to an increased compliance under tensile load with respect to their original counterpart. In similar settings, this biomechanical property appeared to be strongly reduced by GA-treatment (e.g., elastic phase), by confirming previously revealed rigidity of these clinically applied biomaterials [34,55,66]. As generally documented [14,66,67], a similar ability of decellularized bovine pericardium to withstand tensile load as its native counterpart – differently from porcine one – is apparent [55,56,57]. These aptitudes seem to be intrinsically related to a species-specific biomechanical response of analyzed tissues, since they were observed also after the application of different extraction protocols. However, uniaxial tensile testing provides limited information on the biomechanical behavior of investigated tissues, and further assays are needed for a deeper investigation. 

Sulfated GAGs are also essential for many cellular functions [68]. Despite sGAGs removal, no deleterious effects on bioactivity were evidenced in the challenge with two human cells lines, i.e., HUVECs and hBM-MSCs, in terms of proliferation and cytotoxicity, thus confirming the absence of damages to basal membrane proteins. GA-treated pericardium, as applied in several reconstructive surgeries, is known to be poorly cytocompatible [69] and no comprehensive data are available with regard to ISO 10993-5 evaluations for commercial decellularized pericardia already used in the clinics, although declared biocompatible. In most cytocompatibility assays, murine immortalized lines, e.g., NIH/3T3 or L929, or other animals-derived cells are preferred [55,66,67]. In our investigation, we specifically selected human cytotypes to simulate the effects that are exerted by decellularized scaffolds in the *in vivo* human setting. In fact, the reconstitution of a lining by endothelial cells (e.g., HUVECs) onto the scaffold surfaces is extremely relevant in reconstructive applications at the blood interface to prevent thrombotic reactions. Nevertheless, any scaffold has to present a cell-friendly environment, which is permissive of repopulation once implanted, in order to become a living replacement and acquire the ability of self-reparation. In many clinical applications, implanted scaffolds will enter in contact with mesenchymal cells, being mobilized from the bone marrow (i.e., hBM-MSCs) or migrating from anastomotic tissues. In respect to both cells types, DBPs and DPPs induced *in vitro* null cytotoxicity, but an intense proliferation activity. Longer evaluations should be performed to obtain more information on endothelialization and penetration. However, these outcomes are particularly promising for the preservation of the main signals that are involved in the bioactivity of pericardium and, hence, to predict the repopulation of decellularized scaffolds and the successful outcome of a clinical application.

Ideal biomedical materials for implantation surgeries are endowed with characteristics, such as strength, easy molding and suturability, absence of cytotoxicity, biocompatibility, radiolucency, sterile availability, and, not last, inexpensiveness and sustainability [11,22,70]. TRICOL bovine and porcine pericardia, manufactured with a cost-effective decellularization procedure, possess all these features. They can also be provided in optimally sterilized and preserved conditions to/by the tissue banks for their off-the-shelf diffusion in the clinical surgeries [40,71]. 

With regards to the surgical applications, the inferior thickness of the decellularized porcine pericardium is particularly appealing for the fabrication of percutaneous devices, as transcatheter heart valve replacements [55], by minimizing the diameter of the deployment system. We aim at offering novel insights on the bovine and porcine pericardial tissue responses to compressive and expansive loads in an upcoming publication in order to propose more indications for clinical use. For those applications needing resistance to failure under elongation, as in gastrointestinal reconstructive setting, it might be supposed that decellularized bovine pericardium could exhibit a higher failure strain than the porcine one. Table 2 provides hints on the possible clinical applications of TRICOL decellularized bovine and porcine pericardia. After performed analysis, both acellular pericardial scaffolds likely offer similar profiles in terms of structural, biochemical, biomechanical, and bioactivity properties, apart from the differences in thickness, water and GAGs contents. In addition, the comparison hereby performed evidences that also the less clinically applied acellular porcine pericardium could be a biomaterial of interest to be used for surgical reconstruction or replacement, as it is its GA-treated, native counterpart [11].

Although this work is intended to offer a wide-ranging overview of the differential, species-specific properties of the most clinically applied pericardia after their detergent decellularization, other aspects that are relevant for clinical use need to be still investigated. In particular, the analysis of the thrombogenic and inflammatory potentials of these decellularized scaffolds will be object of future studies, possibly relying on *in vitro* and *in vivo* models (e.g., thrombogenicity tests, subcutaneous model, etc.). 

Therefore, with reference to each targeted therapeutic treatment, it appears crucial to carefully consider the species-dependent asset of novel decellularized bioreplacements, as acellular pericardia, before their application.

## 5. Conclusions

This correlative analysis evidenced that TRICOL-decellularized bovine and porcine pericardial scaffolds might be both valued as promising, bioactive materials for reconstruction and replacement purposes. Although they possess a similar regenerative profile, they differ in water content, thickness, and glycosaminoglycans, possibly influencing some of their biomechanical properties and, hence, their indication for surgical use.

## Figures and Tables

**Table 1:** Differences Between Bovine and Porcine Pericardia Before and After TRICOL Decellularization.

		*NBPs vs. NPPs*	*NBPs vs. DBPs*	*NPPs vs. DPPs*	*DBPs vs. DPPs*
*ECM architecture*					
	Serosal collagen C	↓↓↓↓	↓	↓	↓↓↓
	Serosal elastin C	NS	NS	↓↓↓	↓
	Serosal P_s_	NS	NS	NS	↓↓
	Fibrosal collagen C	↓	NS	↓↓↓	↓↓
	Fibrosal elastin C	NS	NS	NS	NS
	Fibrosal P_s_	NS	↑	NS	↓↓↓↓
*ECM biochemical composition*					
	Collagen	NS	↑↑	NS	NS
	Denatured collagen	↓↓↓	NS	NS	↓↓↓↓
	sGAGs	NS	↓↓	NS	NS
	Elastin	NS	NS	NS	NS
	Water	↓↓	↑↑↑↑	↓↓	↓↓↓↓
	DNA	NS	↑↑	↑↑↑	NS
*ECM structure*					
	R	NS	NS	NS	NS
	T_onset_	NS	NS	↓	↓↓↓↓
*ECM biomechanical properties*					
	Thickness	↓↓↓↓	NS	↑↑↑	↓
	Elastin-phase modulus	NS	NS	NS	NS
	Collagen-phase modulus	↑	NS	↓	NS
	Failure strain	↓↓↓	NS	NS	↓
	UTS	NS	NS	NS	NS
*ECM bioactive properties*					
	hBM-MSCs MTS 24 h	/	/	/	NS
	hBM-MSCs MTS 72 h	/	/	/	NS
	HUVECs MTS 24 h	/	/	/	NS
	HUVECs MTS 72 h	/	/	/	NS

Legend: NBP: native bovine pericardium; NPP: native porcine pericardium; DBP: decellularized bovine pericardium; DPP: decellularized porcine pericardium; C: coherence; P_s_: straightness parameter; sGAGs: sulfated glycosaminoglycans; R: ratio between alpha helix and beta-sheet structures; T_onset_: denaturation temperature; UTS: ultimate tensile strength; h-BM-MSCs: human bone marrow-mesenchymal stem cells; HUVECs: human umbilical vein- endothelial cells; MTS: proliferation index; /: not tested; NS: non-significant; ↓ or ↑: *p*< 0.05; ↓↓ or ↑↑: *p*< 0.01; ↓↓↓ or ↑↑↑: *p*< 0.001; ↓↓↓↓ or ↑↑↑↑: *p*< 0.0001.

**Table 2:** Possible Indications for Clinical Applications of TRICOL Decellularized Bovine and Porcine Pericardia.

		*Currently Applied Commercial* *Acellular Animal Pericardia*	*DBPs*	*DPPs*
*Cardiovascular surgery*				
	Heart valve reconstruction, aortic repair, intracardiac defect corrections, annulus repair	Matrix P (E), Photofix (B), CardioCel (B), COVA (B) [26,30,31,32,72,73,74]	+	+
*Gastrointestinal surgery*				
	Anterior abdominal wall reconstruction, hernia repair	Tutopatch (B), Tutomesh (B), COVA (B), Integra (B) [18,19,20,75,76,77]	+*	+
*Maxillofacial, bone and tendon surgeries*				
	Bone reconstruction, Dental reconstruction, Orbital wrapping, Dura mater plastics, Rotor cuff repair, Myringoplasty	Tutopatch (B), Tutoplast (B), Tutodent; CopiOs Pericardium (B); Osteokor pericardium (B); Lyoplant (B); Jason (P) [21,22,23,25,28,29,78,79,80,81]	+**	+
*Plastic surgery*				
	Facial aesthetics, Breast aesthetics	Veritas (B) [27,82]	+	+

Legend: DBP: decellularized bovine pericardium; DPP: decellularized porcine pericardium. Photofix, Cryolife, Kennesaw, GA, US; Matrix P, Auto Tissue Berlin GmbH, Berlin, Germany; CardioCel, Admedus, Geneva, Switzerland; Tutodent, Tutoplast and Tutomesh Tutogen Medical GmbH, Neunkirchen, Germany; COVA, CopiOs Pericardium, Zimmer Biomet Dental, Palm Beach Gardens, FL, US; Lyoplant, Braun, Milan, Italy; Jason ®, Botiss biomaterials, Zossen, Germany; Veritas®, Baxter, Rome Italy; B: bovine; E: equine; P: porcine. + Possible application. * Further analyses are needed to evaluate the suitability of scaffold applications in gastrointestinal reconstructions requiring higher extensibility. ** Further analyses are needed to evaluate the suitability of scaffold applications for tendon surgery.

## Supplementary Materials

The following are available online at https://www.mdpi.com/2218-273X/10/3/371/s1.

## References

[1] Yakirevich V.S., Abdulali S.A., Abbott C.R., Ionescu M.I. (1984). Reconstruction of the pericardial sac with glutaraldehyde-preserved bovine pericardium. Tex Hear. Inst. J..

[2] David T.E., Feindel C.M., Ropchan G.V. (1987). Reconstruction of the left ventricle with autologous pericardium. J. Thorac. Cardiovasc. Surg..

[3] Temeck B.K., Katz N.M., Wallace R.B. (1990). An approach to reoperative median sternotomy. J. Card. Surg..

[4] Grillo H.C. (1994). Slide tracheoplasty for long-segment congenital tracheal stenosis. Ann. Thorac. Surg..

[5] Cribier A., Eltchaninoff H., Bash A., Borenstein N., Tron C., Bauer F., Derumeaux G., Anselme F., Laborde F., Leon M.B. (2002). Percutaneous transcatheter implantation of an aortic valve prosthesis for calcific aortic stenosis: first human case description. Circulation.

[6] Gérard J.M., Gersdorff M. (2006). The Tutopatch graft for transcanal myringoplasty. B-ENT.

[7] Stavropoulos A., Chiantella G., Costa D., Steigmann M., Windisch P., Sculean A. (2011). Clinical and Histologic Evaluation of a Granular Bovine Bone Biomaterial Used as an Adjunct to GTR With a Bioresorbable Bovine Pericardium Collagen Membrane in the Treatment of Intrabony Defects. J. Periodontol..

[8] Merli M., Moscatelli M., Mariotti G., Pagliaro U., Raffaelli E., Nieri M. (2015). Comparing membranes and bone substitutes in a one-stage procedure for horizontal bone augmentation. A double-blind randomised controlled trial. Eur. J. Oral Implantol..

[9] Ionescu M.I., Pakrashi B.C., Holden M.P., Mary D.A., Wooler G.H. (1972). Results of aortic valve replacement with frame-supported fascia lata and pericardial grafts. J. Thorac. Cardiovasc. Surg..

[10] Manji R.A., Zhu L.F., Nijjar N.K., Rayner D.C., Korbutt G.S., Churchill T.A., Rajotte R.V., Koshal A., Ross D.B. (2006). Glutaraldehyde-fixed bioprosthetic heart valve conduits calcify and fail from xenograft rejection. Circulation.

[11] Iop L., Palmosi T., Dal Sasso E., Gerosa G. (2018). Bioengineered tissue solutions for repair, correction and reconstruction in cardiovascular surgery. J. Thorac. Dis..

[12] Courtman D.W., Pereira C.A., Kashef V., McComb D., Lee J.M., Wilson G.J. (1994). Development of a pericardial acellular matrix biomaterial: biochemical and mechanical effects of cell extraction. J. Biomed. Mater. Res..

[13] Mirsadraee S., Wilcox H.E., Korossis S.A., Kearney J.N., Watterson K.G., Fisher J., Ingham E. (2006). Development and Characterization of an Acellular Human Pericardial Matrix for Tissue Engineering. Tissue Eng..

[14] Hülsmann J., Grün K., El Amouri S., Barth M., Hornung K., Holzfuß C., Lichtenberg A., Akhyari P. (2012). Transplantation material bovine pericardium: Biomechanical and immunogenic characteristics after decellularization vs. glutaraldehyde-fixing. Xenotransplantation.

[15] Cigliano A., Gandaglia A., Lepedda A.J., Zinellu E., Naso F., Gastaldello A., Aguiari P., De Muro P., Gerosa G., Spina M. (2012). Fine Structure of Glycosaminoglycans from Fresh and Decellularized Porcine Cardiac Valves and Pericardium. Biochem. Res. Int..

[16] Aguiari P., Iop L., Favaretto F., Fidalgo C.M.L., Naso F., Milan G., Vindigni V., Spina M., Bassetto F., Bagno A. (2017). In vitro comparative assessment of decellularized bovine pericardial patches and commercial bioprosthetic heart valves. Biomed. Mater..

[17] Etnel J.R.G., Suss P.H., Schnorr G.M., Veloso M., Colatusso D.F., Balbi Filho E.M., da Costa F.D.A. (2018). Fresh decellularized versus standard cryopreserved pulmonary allografts for right ventricular outflow tract reconstruction during the Ross procedure: A propensity-matched study. Eur. J. Cardio-Thoracic Surg..

[18] Tutopatch Tutopatch TM and Tutomesh TM Bovine Pericardium Implant Overview. http://www.lifehealthcare.com.au/wp-content/uploads/2017/07/Tutomesh_and_Tutopatch_Comprehensive_Implant_Overview.pdf.

[19] COVA+ABDO COVATM+ ABDO. www.biomup.com/en/cova-plus/cova-plus-abdo/3/.

[20] INTEGRA Flexibility in Your Hands INTEGRA BP Flexible Performance Integra BP: Sutured Dural Graft Easily Conforms to Protect Against CSF Leaks Integra ® Bovine Pericardium (BP) Dural Graft. www.integralife.com/file/general/1478293848.pdf.

[21] OSTEOKOR Osteokor Pericardium. https://www.surgikorimplants.com/regeneration/osteokor-pericardium.

[22] Filippi R., Schwarz M., Voth D., Reisch R., Grunert P., Perneczky A. (2001). Bovine pericardium for duraplasty: clinical results in 32 patients. Neurosurg. Rev..

[23] Steigmann M. (2006). Pericardium Membrane and Xenograft Particulate Grafting Materials for Horizontal Alveolar Ridge Defects. Implant Dent..

[24] Hoell T., Hohaus C., Huschak G., Beier A., Meisel H.-J. (2007). Total dura substitute in the spine: double layer dural substitute made from polylactide layer and bovine pericardium. Acta Neurochir..

[25] Albera R., Dagna F., Lacilla M., Canale A. (2009). Equine versus Bovine Pericardium in Transmeatal Underlay Myringoplasty. Ann. Otol. Rhinol. Laryngol..

[26] Bel A., Kachatryan L., Bruneval P., Peyrard S., Gagnieu C., Fabiani J.-N., Menasché P. (2010). A new absorbable collagen membrane to reduce adhesions in cardiac surgery. Interact. Cardiovasc. Thorac. Surg..

[27] Gubitosi A., Docimo G., Parmeggiani D., Pirozzi R., Vitiello C., Schettino P., Avellino M., Casalino G., Amato M., Ruggiero R. (2014). Acellular bovine pericardium dermal matrix in immediate breast reconstruction after Skin Sparing Mastectomy. Int. J. Surg..

[28] de Dorlodot C., De Bie G., Deggouj N., Decat M., Gérard J.-M. (2015). Are bovine pericardium underlay xenograft and butterfly inlay autograft efficient for transcanal tympanoplasty?. Eur. Arch. Oto-Rhino-Laryngol..

[29] Le B., Borzabadi-Farahani A., Nielsen B. (2016). Treatment of Labial Soft Tissue Recession Around Dental Implants in the Esthetic Zone Using Guided Bone Regeneration With Mineralized Allograft: A Retrospective Clinical Case Series. J. Oral Maxillofac. Surg..

[30] PhotoFix PhotoFix Decellularized Bovine Pericardium. www.cryolife.com.

[31] Majeed A., Baird C., Borisuk M.J., Sanders S.P., Padera R.F. (2016). Histology of Pericardial Tissue Substitutes Used in Congenital Heart Surgery. Pediatr. Dev. Pathol..

[32] Matrix Patch The Equine Matrix Patch TM is a Cell-Free Pericardial Patch for Use in Cardiac Surgery. www.autotissue.de/fileadmin/user_upload/Flyer_neu_Homepage.pdf.

[33] EACTS Satellite symposium 2017 5 Years Experience with the Decellularized Matrix Patch—Auto Tissue Satellite Symposium-Monday-EACTS. www.eacts.org/educational-events/eacts-annual-meeting/scientific-programme/auto-tissue-satellite-symposium-monday/.

[34] Gauvin R., Marinov G., Mehri Y., Klein J., Li B., Larouche D., Guzman R., Zhang Z., Germain L., Guidoin R. (2013). A comparative study of bovine and porcine pericardium to highlight their potential advantages to manufacture percutaneous cardiovascular implants. J. Biomater. Appl..

[35] Bagno A., Aguiari P., Fiorese M., Iop L., Spina M., Gerosa G. (2017). Native Bovine and Porcine Pericardia Respond to Load With Additive Recruitment of Collagen Fibers. Artif. Organs.

[36] Iop L., Renier V., Naso F., Piccoli M., Bonetti A., Gandaglia A., Pozzobon M., Paolin A., Ortolani F., Marchini M. (2009). The influence of heart valve leaflet matrix characteristics on the interaction between human mesenchymal stem cells and decellularized scaffolds. Biomaterials.

[37] Gallo M., Naso F., Poser H., Rossi A., Franci P., Bianco R., Micciolo M., Zanella F., Cucchini U., Aresu L. (2012). Physiological Performance of a Detergent Decellularized Heart Valve Implanted for 15 Months in Vietnamese Pigs: Surgical Procedure, Follow-up, and Explant Inspection. Artif. Organs.

[38] Iop L., Bonetti A., Naso F., Rizzo S., Cagnin S., Bianco R., Dal Lin C., Martini P., Poser H., Franci P. (2014). Decellularized allogeneic heart valves demonstrate self-regeneration potential after a long-term preclinical evaluation. PLoS One.

[39] Iop L., Paolin A., Aguiari P., Trojan D., Cogliati E., Gerosa G. (2017). Decellularized Cryopreserved Allografts as Off-the-Shelf Allogeneic Alternative for Heart Valve Replacement: In Vitro Assessment Before Clinical Translation. J. Cardiovasc. Transl. Res..

[40] Fidalgo C., Iop L., Sciro M., Harder M., Mavrilas D., Korossis S., Bagno A., Palù G., Aguiari P., Gerosa G. (2018). A sterilization method for decellularized xenogeneic cardiovascular scaffolds. Acta Biomater..

[41] Sacks M.S., Chuong C.J.C., More R. (1994). Collagen fiber architecture of bovine pericardium. ASAIO J..

[42] Filippi A., Dal Sasso E., Iop L., Armani A., Gintoli M., Sandri M., Gerosa G., Romanato F., Borile G. (2018). Multimodal label-free ex vivo imaging using a dual-wavelength microscope with axial chromatic aberration compensation. J. Biomed. Opt..

[43] OrientationJ. http://bigwww.epfl.ch/demo/orientation/.

[44] Schindelin J., Arganda-Carreras I., Frise E., Kaynig V., Longair M., Pietzsch T., Preibisch S., Rueden C., Saalfeld S., Schmid B. (2012). Fiji: An open-source platform for biological-image analysis. Nat. Methods.

[45] Rezakhaniha R., Agianniotis A., Schrauwen J.T., Griffa A., Sage D., Bouten C.V., van de Vosse F.N., Unser M., Stergiopulos N. (2012). Experimental investigation of collagen waviness and orientation in the arterial adventitia using confocal laser scanning microscopy. Biomech. Model. Mechanobiol..

[46] NeuronJ. https://omictools.com/neuronj-tool.

[47] Bank R.A., Krikken M., Beekman B., Stoop R., Maroudas A., Lafebers F.P.J.G., Te Koppele J.M. (1997). A simplified measurement of degraded collagen in tissues: Application in healthy, fibrillated and osteoarthritic cartilage. Matrix Biol..

[48] Edwards C.A., O’Brien W.D. (1980). Modified assay for determination of hydroxyproline in a tissue hydrolyzate. Clin. Chim. Acta.

[49] Korossis S.A., Booth C., Wilcox H.E., Watterson K.G., Kearney J.N., Fisher J., Ingham E. (2002). Tissue engineering of cardiac valve prostheses II: biomechanical characterization of decellularized porcine aortic heart valves. J. Heart Valve Dis..

[50] ISO 10993-5 ISO 10993-5 Biological Evaluation of Medical Devices - Part 5: Tests for In Vitro Cytotoxicity. www.iso.org/standard/68936.html.

[51] Cebotari S., Tudorache I., Jaekel T., Hilfiker A., Dorfman S., Ternes W., Haverich A., Lichtenberg A. (2010). Detergent decellularization of heart valves for tissue engineering: toxicological effects of residual detergents on human endothelial cells. Artif Organs.

[52] Motulsky H.J., Brown R.E. (2006). Detecting outliers when fitting data with nonlinear regression – a new method based on robust nonlinear regression and the false discovery rate. BMC Bioinformatics.

[53] Benjamini Y., Krieger A.M., Yekutieli D. (2006). Adaptive linear step-up procedures that control the false discovery rate. Biometrika.

[54] Holzapfel G.A. (2003). Structural and numerical models for the (visco)elastic response of arterial walls with residual stresses. Biomechanics of Soft Tissue in Cardiovascular Systems.

[55] Choe J.A., Jana S., Tefft B.J., Hennessy R.S., Go J., Morse D., Lerman A., Young M.D. (2018). Biomaterial characterization of off-the-shelf decellularized porcine pericardial tissue for use in prosthetic valvular applications. J. Tissue Eng. Regen. Med..

[56] Mendoza-Novelo B., Avila E.E., Cauich-Rodríguez J.V., Jorge-Herrero E., Rojo F.J., Guinea G.V., Mata-Mata J.L. (2011). Decellularization of pericardial tissue and its impact on tensile viscoelasticity and glycosaminoglycan content. Acta Biomater..

[57] Morticelli L., Thomas D., Ingham E., Korossis S. (2012). Investigation of the Suitability of Decellularised Porcine Pericardium for Mitral Valve Reconstruction. QScience Proc..

[58] Sasisekharan R., Raman R., Prabhakar V. (2006). Glycomics approach to structure-function relationships of glycosaminoglycans. Annu. Rev. Biomed. Eng..

[59] Gesslbauer B., Rek A., Falsone F., Rajkovic E., Kungl A.J. (2007). Proteoglycanomics: tools to unravel the biological function of glycosaminoglycans. Proteomics.

[60] Kozlowski E.O., Pavao M.S.G., Borsig L. (2011). Ascidian dermatan sulfates attenuate metastasis, inflammation and thrombosis by inhibition of P-selectin. J. Thromb. Haemost..

[61] Sobczak A.I.S., Pitt S.J., Stewart A.J. (2018). Glycosaminoglycan Neutralization in Coagulation Control. Arterioscler. Thromb. Vasc. Biol..

[62] Rabinovich G.A., van Kooyk Y., Cobb B.A. (2012). Glycobiology of immune responses. Ann. N. Y. Acad. Sci..

[63] Langford-Smith A., Day A.J., Bishop P.N., Clark S.J. (2015). Complementing the Sugar Code: Role of GAGs and Sialic Acid in Complement Regulation. Front. Immunol..

[64] Raspanti M., Viola M., Forlino A., Tenni R., Gruppi C., Tira M.E. (2008). Glycosaminoglycans show a specific periodic interaction with type I collagen fibrils. J. Struct. Biol..

[65] Mizumoto S., Kosho T., Yamada S., Sugahara K. (2017). Pathophysiological Significance of Dermatan Sulfate Proteoglycans Revealed by Human Genetic Disorders. Pharmaceuticals.

[66] Bai M., Zhang T., Ling T., Zhou Z., Xie H., Zhang W., Hu G., Jiang C., Li M., Feng B. (2014). Guided bone regeneration using acellular bovine pericardium in a rabbit mandibular model: in-vitro and in-vivo studies. J. Periodontal Res..

[67] Li N., Li Y., Gong D., Xia C., Liu X., Xu Z. (2018). Efficient decellularization for bovine pericardium with extracellular matrix preservation and good biocompatibility. Interact. Cardiovasc. Thorac. Surg..

[68] Mikami T., Kitagawa H. (2017). Sulfated glycosaminoglycans: their distinct roles in stem cell biology. Glycoconj. J..

[69] Petite H., Duval J.L., Frei V., Abdul-Malak N., Sigot-Luizard M.F., Herbage D. (1995). Cytocompatibility of calf pericardium treated by glutaraldehyde and by the acyl azide methods in an organotypic culture model. Biomaterials.

[70] Williams D.F. (2019). Challenges With the Development of Biomaterials for Sustainable Tissue Engineering. Front. Bioeng. Biotechnol..

[71] Zouhair S., Aguiari P., Iop L., Vásquez-Rivera A., Filippi A., Romanato F., Korossis S., Wolkers W.F., Gerosa G. (2018). Preservation strategies for decellularized pericardial scaffolds for off-the-shelf availability. Acta Biomater..

[72] Neethling W.M.L., Strange G., Firth L., Smit F.E. (2013). Evaluation of a tissue-engineered bovine pericardial patch in paediatric patients with congenital cardiac anomalies: Initial experience with the ADAPT-treated CardioCel® patch. Interact. Cardiovasc. Thorac. Surg..

[73] Sobieraj M., Cudak E., Mrówczyński W., Nałęcz T.K., Westerski P., Wojtalik M. (2016). Application of the CardioCel bovine pericardial patch - a preliminary report. Kardiochirurgia i torakochirurgia Pol. = Polish J. cardio-thoracic Surg..

[74] De Martino A., Milano A.D., Bortolotti U. (2019). Use of Pericardium for Cardiac Reconstruction Procedures in Acquired Heart Diseases—A Comprehensive Review. Thorac. Cardiovasc. Surg..

[75] Smart N.J., Marshall M., Daniels I.R. (2012). Biological meshes: A review of their use in abdominal wall hernia repairs. Surgeon.

[76] Sun L., Chen J., Li J., Shen Y. (2019). Randomized and Comparative Clinical Trial of Bovine Mesh Versus Polypropylene Mesh in the Repair of Inguinal Hernias. Surg. Laparosc. Endosc. Percutaneous Tech..

[77] Costa A., Adamo S., Gossetti F., D’Amore L., Ceci F., Negro P., Bruzzone P. (2019). Biological scaffolds for abdominal wall repair: Future in clinical application?. Materials.

[78] Ono Y., Dávalos Herrera D.A., Woodmass J.M., Boorman R.S., Thornton G.M., Lo I.K.Y. (2016). Can Grafts Provide Superior Tendon Healing and Clinical Outcomes After Rotator Cuff Repairs?. Orthop. J. Sport. Med..

[79] Gayre G.S., Debacker C., Lipham W., Tawfik H.A., Holck D., Dutton J.J. (2001). Bovine pericardium as a wrapping for orbital implants. Ophthal. Plast. Reconstr. Surg..

[80] Straumann Jason. https://www.straumann.com/en/dental-professionals/products-and-solutions/biomaterials/membranes.html.

[81] Giesenhagen B., Martin N., Jung O., Barbeck M. (2019). Bone augmentation and simultaneous implant placement with allogenic bone rings and analysis of its purification success. Materials.

[82] Mofid M.M., Meininger M.S., Lacey M.S. (2012). Veritas® bovine pericardium for immediate breast reconstruction: a xenograft alternative to acellular dermal matrix products. Eur. J. Plast. Surg..

