# Supplementary material for: A Comprehensive Comparison of Bovine and Porcine Decellularized Pericardia: New Insights for Surgical Applications
Source: Biomolecules. 2020 Feb 28;10(3):371. doi: 10.3390/biom10030371 (PMC7175169; doi:10.3390/biom10030371)
Supplement: Supplementary file 1 [file biomolecules-10-00371-s001.pdf]

Supplementary Material

A Comprehensive Comparison of Bovine and Porcine Decellularized Pericardia: New Insights for Surgical Applications

Sabra Zouhair [1,+], Eleonora Dal Sasso [1,+], Sugat R. Tuladhar [1,+], Catia Fidalgo [1], Luca Vedovelli [2], Andrea Filippi [3,4,5], Giulia Borile [3,5,6], Andrea Bagno [7,8], Massimo Marchesan [1], De Rossi Giorgio [9], Dario Gregori [2], Willem F. Wolkers [10], Filippo Romanato [3,5,8,11], Sotirios Korossis [12,13,14], Gino Gerosa [1,8,§], and Laura Iop [1,8,§,*]

[1] Cardiovascular Regenerative Medicine, Department of Cardiac Thoracic Vascular Sciences and Public Health, University of Padua, I-35128 Padua, Italy
[2] Biostatistics, Department of Cardiac Thoracic Vascular Sciences and Public Health, University of Padua, I-35128 Padua, Italy
[3] Department of Physics and Astronomy "G. Galilei," University of Padua, I-35131 Padua, Italy
[4] Fondazione Bruno Kessler, I-38123 Trento, Italy,
[5] Institute of Pediatric Research Città della Speranza, I-35127 Padua, Italy
[6] Department of Biomedical Sciences, University of Padua, I-35131 Padua, Italy
[7] Department of Industrial Engineering, University of Padua, I-35131 Padua, Italy
[8] L.I.F.E.L.A.B. Program, Consorzio per la Ricerca Sanitaria (CORIS), Veneto Region, I-35127 Padua, Italy
[9] ULSS 3 Serenissima, Mestre, I-30174 Venice, Italy
[10] Institute of Multiphase Processes, Leibniz Universität Hannover, D-30167 Hannover, Germany
[11] Laboratory for Nanofabrication of Nanodevices, I-35127 Padua, Italy
[12] Department of Cardiothoracic, Transplantation and Vascular Surgery, Hannover Medical School, D-30625 Hannover, Germany
[13] Lower Saxony Centre for Biomedical Engineering Implant Research and Development, Hannover Medical School, D-30625 Hannover, Germany
[14] Centre for Biological Engineering, Wolfson School of Mechanical, Electrical and Manufacturing Engineering, Loughborough University, LE11 3TU Loughborough, Leicestershire, United Kingdom.
[+] These authors contributed equally.
[§] These authors contributed equally.
* Correspondence: laura.iop@unipd.it; Tel.: +39-049-821-7651

Received: 23 January 2020; Accepted: 17 February 2020; Published: date

Table 1 Quantification of ECM Components, Water and DNA Contents in Native and Decellularized Pericardia

	NBPs	*DBPs*	*p*	*NPPs*	*DPPs*	*p*
HYP ($\mu g\ mg^{-1}$)	106.60 ± 3.11	113.26 ± 1.96	0.0063	114.48 ± 11.18	121.30 ±11.38	0.3667
Denatured HYP ($\mu g\ mg^{-1}$)	12.06 ± 6.74	16.66±8.18	0.9949	5.85±1.50	4.93±1.14	0.5435
sGAGs ($\mu g\ mg^{-1}$)	12.59 ± 4.10	5.75 ± 1.01	0.0085	8.15 ± 2.49	6.15 ± 1.01	0.1145
Elastin ($\mu g\ mg^{-1}$)	44.95 ± 11.12	48.88 ± 18.36	0.6634	49.55 ± 6.58	46.42 ± 11.78	0.5828
Water (%)	81.01 ± 0.87	83.00 ± 00.00	< 0.0001	78.36 ± 2.67	75.26 ± 0.95	0.0031
DNA ($\mu g\ mg^{-1}$)	1446 ± 699.8	66.05 ± 72.79	0.0046	1774 ± 385.8	56.47 ± 40.11	0.0001

Legend: NBP: native bovine pericardium; DBP: decellularized bovine pericardium; NPP: native porcine pericardium; DPP: decellularized porcine pericardium; HYP: hydroxyproline; sGAGs: sulfated glycosaminoglycans.
